# Gracilariopsis persica from Persian Gulf Contains Bioactive Sterols

**Published:** 2012

**Authors:** Soodabeh Saeidnia, Parisa Permeh, Ahmad Reza Gohari, Ali Mashinchian-Moradi

**Affiliations:** a *Medicinal Plants Research Center, Faculty of Pharmacy, Tehran University of Medical Sciences, Tehran, Iran.*; b *Department of Marine Science and Technology, Sciences and Research Center, Islamic Azad University, Tehran, Iran.*

**Keywords:** *Gracilariopsis Persica*, 22-dehydrocholesterol, Cholesterol, Fucosterol, Cytotoxicity

## Abstract

*Gracilariopsis Persica *(Rhodophyta) is one of the most abundant algae, introduced newly from the Indian Ocean. In this study, the main sterols of the algae have been isolated and identified. Separation and purification of the compounds was carried out on silica gel and sephadex LH_20_ column chromatography (CC) and high performance liquid chromatography (HPLC) to obtain five pure compounds 1-5. Structural elucidation of the compounds was based on the data obtained from H-NMR, ^13^C-NMR, DEPT and Mass spectroscopy. The separated compounds from *Gp. Persica *were identified as 22-dehydrocholesterol (1), cholesterol (2), stigmasterol (3), *β*-sitosterol (4) and fucosterol (5) based on the spectral data compared to those reported in literatures. Most of these sterols are noteworthy for their effectiveness in decreasing the plasma cholesterol, glucose and inflammation. The results of Brine Shrimp Cytotoxicity Assay indicated that the ethyl acetate extract of *Gp. Persica *showed a high cytotoxic effect against *A. salina *nauplii (LC_50_ = 4 μg/mL). The methanol extract was no effective but the aqueous methanol extract was moderately effective (LC_50 _= 40 μg/mL) compared to berberine hydrochloride as a positive control (LC_50_ = 26 μg/mL).

## Introduction

*Gracilariopsis persica *(Rhodophyceae), introduced newly from the Indian Ocean, belongs to the Gracilariaceae family as a difficult group over decades (1). So far, various sterols were separated and identified from the red algae. Among them, desmosterol was averaged 73.6% of the total sterol in twenty-three samples and cholesterol was the second principal sterol averaging 13%. The sterol composition was a variable independent of the location or time of the collection (2). The Gracilariaceae (Rhodophyta) has emerged as one of the families that possess economic potential as a source of agar and as a potential feed for abalone (3). *Gracilariopsis *is a considerable genus in Gracilariaceae family since approximately all of its species include the highly conserved gross and reproductive morphology, very similar to *Gp. Andersonii *and difficult to distinguish (4).

*Gracilariopsis persica *is one of the most abundant red algae distributed in Persian Gulf, which there is no literature around its sterol composition. In this paper, the isolation and structural elucidation of the sterols from the ethyl acetate and methanol extracts of these marine algae are explained for the first time.

## Experimental

*Instruments and reagents*

^1^H- and ^13^C-NMR spectra were measured on a Bruker Avance 500 DRX (500 MHz for ^1^H and 125 MHz for ^13^C) spectrometer with tetramethylsilane as an internal standard and chemical shifts are given in δ (ppm). EI-MS data were recorded on Agilent Technology (HP) instrument with 5973 Network Mass Selective Detector (MS model). Silica gel 60F_254_ pre-coated plates (Merck) were used for TLC. The spots were detected through spraying anisaldehyde-H_2_SO_4_ reagent followed by heating (120 ºC for 5 min).

*Algae material*

Red algae was collected from the northern areas of Persian Gulf in July (2008) and identified as *Gracilariopsis persica *sp. Nov. This is a tetrasporangial algae from Iran, Persian Gulf, Bandarabbas (27˚10’ N, 58˚16’ E). A voucher specimen (64-27R) has been deposited at the Herbarium of Persian Gulf Biotechnology Research Center (Gheshm Island).

*Extractions of the marine algae*

Marine algae were dried carefully and cut into small pieces. Dried powder of algae (1126 g) was extracted with ethyl acetate, methanol and aqueous methanol (50%), respectively, via percolation (72 h each time) at room temperature. Then, the solvents evaporated under reduced pressure and were dried under the vacuum in order to give dried powder of the ethyl acetate (2 g), methanol (54 g) and aqueous methanol (5 g) extracts.

*Isolation of the sterols*

The methanol extract (50 g) was submitted to the silica gel column chromatography (CC) with CHCl_3_ : AcOEt (19 : 1, 7 : 3, 0 : 1), MeOH and aqueous methanol (8%), consequently, to obtain 6 fractions (A-F). The fraction B (2.3 g) was subjected to silica gel CC, with Hexane : AcOEt (9 : 1) to give three fractions (B_1_-B_3_). The fraction, B_2_ (3.5 mg), was the mixture of compounds 1 and 2 (3.5 mg). The fraction C (259 g) was subjected to silica gel CC, with Hexane : AcOEt (9 : 1) to give two fractions (C_1_ and C_2_). The fraction, C_1_ (46 mg), was the pure compound 1 (46 mg). The fraction E (2.5 g) was subjected to sephadex LH_20_ with MeOH : CHCl_3_ (7 : 3) to gain four sub fractions (E_1_-E_4_). Fraction E_1_ (80 mg) was submitted to the HPLC (Knauer). HPLC conditions are as follows: Vertex column C18 (250 × 20 mm I.D.), Kenauer. Gradient elution: 0.00 min 50% MeOH, 15.00 min 100% MeOH until 50.00 min. Flow-rate: 3 mL min^-1^. The injection volume and the detector were 2 mL and PDA (UV-spectra were collected across the range of 200-900 nm). The wave length was 210 nm for detecting the compounds. Nine fractions (E_11_-E_19_) were obtained using HPLC, of which, the fractions E_12_ and E_16_ were the pure compounds 3 (4 mg, Rt = 33.10 min) and 4 (2 mg, Rt = 35.06 min), respectively. Fraction F (1.2 g) was submitted to sephadex LH_20_ with MeOH to obtain seven sub fractions (F_1_-F_7_). The fraction F_4_ (3 mg), was the pure compound 5.

*Brine shrimp cytotoxicity bioassay (BSC)*

Brine shrimp (*Artemia salina*) eggs were purchased from the Shilat Center (Tehran). The eggs were hatched in a flask containing 300 mL of artificial seawater. The flask was well aerated with the aid of an air pump and kept in a water bath at 29-30 ºC. A bright light was left on the nauplii hatched within 48 h. The extracts (ethyl acetate, methanol and aqueous methanol) were dissolved in normal saline. Different concentrations were obtained through serial dilution. Solution of each concentration (500 μL) was transferred into 24-well plates through a pipette and aerated seawater having 10-20 nauplii (500 μL) was added. A check count was performed and the number alive were noted after 24 h. The mortality end point of the bioassay was determined as the absence of controlled forward motion during 30 sec of observation. The controls used were seawater and berberine hydrochloride (LC_50 _= 26 μg/mL). The lethality percentage was determined and LC_50_ was calculated based on the Probit Analysis with 95% of confidence interval (5).

## Results and Discussion

**Figure 1:**
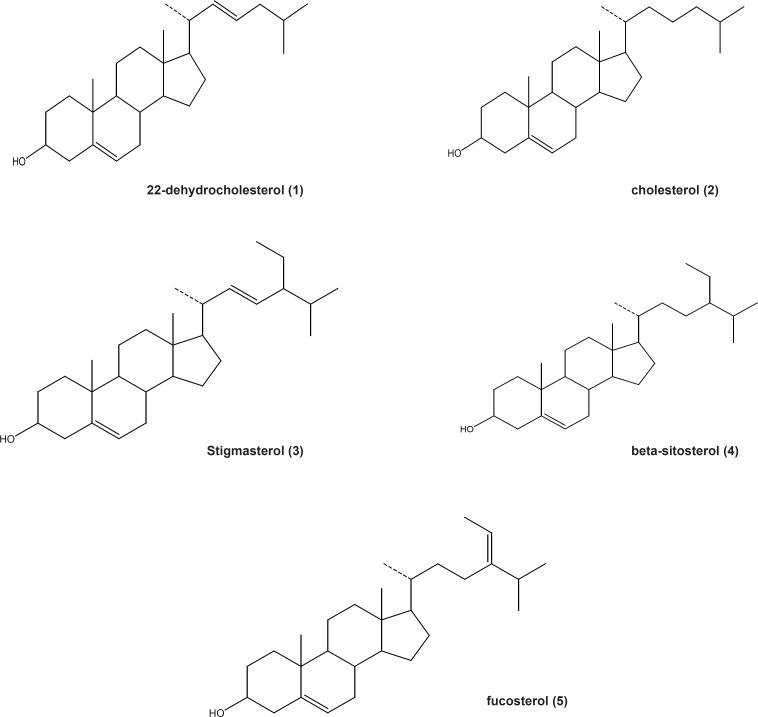
*Structures of the sterols isolated from *Gp.persica

MeOH extract of *Gp. persica*, gathered from Persian Gulf, have been used for the separation of sterols. Isolation and purification of the main compounds was carried out on silica gel, sephadex LH_20_ CC and HPLC to obtain five pure compounds (1-5). Structural elucidation of the constituents was based on the data obtained from H-NMR, ^13^C-NMR, DEPT and Mass spectroscopy. The separated compounds from *Gp. Persica *were identified as 22-dehydrocholesterol (1), cholesterol (2), stigmasterol (3), *β*-sitosterol (4) and fucosterol (5) (Figure 1) based on the spectral data compared to those reported in literatures (6-8).

The results showed that cholesterol is one of the main sterols followed by 22-dehydrocholesterol in *Gp. Persica*. Literature review indicated that most of the red algae (Rhodophyta) contain primarily cholesterol (9). The concentration of cholesterol, the typical sterol in red algae, is also significant in brown algae. Fucosterol, another characteristic sterol for brown algae, has been isolated from *Gp. Persica *too (8). Fucosterol has been reported for its anti-diabetic activity. The administration of fucosterol (orally) at 30 mg/Kg in streptozotocin-induced diabetic rats produced a significant decrease in serum glucose concentrations. In addition, the administration of fucosterol (300 mg/Kg) in epinephrine-induced diabetic rats inhibited the blood glucose level and glycogen degradation (10). Therefore, fucosterol could be a main anti-diabetic principle in the marine algae *Gp. Persica*. Besides, fucosterol has been reported to be an anti-oxidative sterol from the marine algae, *Pelvetia siliquosa *through increasing the anti-oxidant enzymes (11). Stigmasterol and *β*-sitosterol are two of the main sterols distributed widely in plant kingdom but traces in the red algae. Plant sterols compete with cholesterol for intestinal absorption to limit the absorption and lower plasma concentrations of cholesterol. Stigmasterol, but not *β*-sitosterol, has been reported to inhibit cholesterol biosynthesis via the inhibition of sterol Delta (24)-reductase in human Caco-2 and HL-60 cell lines (12). Stigmasterol consumption (orally) inhibits intestinal cholesterol and other plant sterol absorption and suppresses the hepatic cholesterol and classic bile acid synthesis in Wistar rats (12). The compound *β*-sitosterol is reported to reduce the symptoms of Benin Prostatic Hyperplasia (BPH) and also found as an anti-inflammatory agent (13, 14).

The results of Brine Shrimp Cytotoxicity Assay indicated that the ethyl acetate extract of *Gp. Persica *showed a high cytotoxic effect against *A. salina *nauplii (LC_50_ = 4 μg/mL). The MeOH extract of *Gp. Persica *showed no activity but the aqueous methanol extract was less effective (LC_50_ = 40 μg/mL) compared to berberine hydrochloride as a positive control (LC_50_ = 26 μg/mL). Previously, we reported the toxicity of ethyl acetate extracts of two red marine algae, *Gracilaria salicornia *and *Hypnea flagelliformis *(15)*. *The brine shrimp lethality of the ethyl acetate extract of *Gp. Persica *seems to be due to the presence of stigmasterol and *β*-sitosterol which were interestingly reported to be toxic against the brine shrimps (16).

In conclusion, the results of this study show that the red marine algae *Gp. Persica*, which has been abundantly cultured for the animal feeding and other industrial purposes, is rich of sterols. Among the main identified sterols, fucosterol, stigmasterol and *β*-sitosterol are noteworthy for their effectiveness in decreasing the plasma cholesterol, glucose and inflammation.
